# Membrane engineering of cell membrane biomimetic nanoparticles for nanoscale therapeutics

**DOI:** 10.1002/ctm2.292

**Published:** 2021-01-21

**Authors:** Minghai Zhang, Shanshan Cheng, Yue Jin, Nan Zhang, Yu Wang

**Affiliations:** ^1^ Department of Obstetrics and Gynecology, Renji Hospital, School of Medicine Shanghai Jiaotong University Shanghai China; ^2^ Shanghai Key Laboratory of Gynecologic Oncology Shanghai China

**Keywords:** cell membrane camouflaged nanoparticles, membrane engineering, nanoscale therapeutics

## Abstract

In recent years, cell membrane camouflaging technology has emerged as an important strategy of nanomedicine, and the modification on the membranes is also a promising approach to enhance the properties of the nanoparticles, such as cancer targeting, immune evasion, and phototherapy sensitivity. Indeed, diversified approaches have been exploited to re‐engineer the membranes of nanoparticles in several studies. In this review, first we discuss direct modification strategy of cell membrane camouflaged nanoparticles (CM‐NP) via noncovalent, covalent, and enzyme‐involved methods. Second, we explore how the membranes of CM‐NPs can be re‐engineered at the cellular level using strategies such as genetic engineering and membranes fusion. Due to the innate biological properties and excellent biocompatibility, the functionalized cell membrane‐camouflaged nanoparticles have been widely applied in the fields of drug delivery, imaging, detoxification, detection, and photoactivatable therapy.

## Graphical abstract

Cell membrane biomimetic nanoparticles nowadays are developed rapidly. But the cell membrane coats have their inherent limitation, such as the deficiency of targeting ability. To endow additional ability to the cell membrane coated nanoparticle, the modification on the membrane seems a promising strategy.

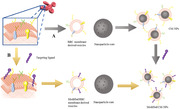

## 1 INTRODUCTION

With the improvement of nanotechnologies, the nanoparticles have been widely applied in the cancer therapy.^1^, ^2^, ^3^, ^4^, ^5^ The enhanced permeation and retention (EPR) effect, which means the selective accumulation and existence of nanoparticles and polymeric medicines in solid tumors compared to normal tissues, has been observed since 1980s.^6^ Owing to the EPR effect, the nanotechnology has been considered to be an appropriate platform to synthetize anti‐tumor drug delivers.^7^ The nanoparticles are promising materials targeting to the desired cancer cells,^8^, ^9^, ^10^ but the preparation of functional nanoparticles is full of challenges. The synthetic nanoparticles are easily interfered by an immense range of proteins expressed on varied cytomembranes in vivo.^11^, ^12^ To add extra properties to the nanoplatforms, polymer materials have been utilized to functionalize the surface of nanoparticles.^13^, ^14^, ^15^, ^16^ However, with the poor biocompatibility and antigenicity, the polymer surfaces activate the immune response and tend to be attached by serum proteins, thus forming the “protein corona”.^17^, ^18^, ^19^, ^20^ The “protein corona” leads to the loss of polymers’ function.

Cells, the most fundamental units of organisms, can manage to survive and carry out the specific functions in the environment containing a large range of proteins, extracellular matrices, and various cells. Researchers intend to retain the unbelievable sensitivity and specificity in nature. Given the shortages of polymer materials, recently a novel biomimetic nanoplatform exploiting natural cell membrane as the cloaks of nanoparticles has emerged.^2^, ^6^, ^21^, ^22^, ^23^ The membrane camouflaging technology was first proposed in 2011, in which researchers utilized erythrocytes as the source of membrane materials.^22^ Unlike polymer materials, cell membranes are natural materials of organisms with a great advantage of biocompatibility. The cell membrane coated nanoparticles (CM‐NPs) possess innate varieties because the coating membrane can derive from any cells, such as erythrocytes, platelets, cancer cells, stem cells, immunocytes, and even bacteria.^24^, ^25^ CM‐NPs combine the merits of natural and synthetic nanomaterials. Cell membrane coating can inherit the characteristics of source cells, such as tumor targeting and long circulation.

Although the technology of cytomembranes with biological abilities as cloaks of nano‐formulations has a relatively dominant position in severe fields, including targeted drug delivery, enhancing immunocompetence, detecting cancerous cells, photodynamic therapy (PDT) and photothermal therapy (PTT), it is inevitable that the function of natural cytomembranes has certain limitations.^6^, ^26^, ^27^, ^28^, ^29^ For example, CM‐NPs derived from erythrocytes have a deficiency of identifying cancer cells, and the targeting abilities are not intense enough.^6^ To solve these problems, there are great demands for reforming cell membranes to adapt to practical applications. It is conceivable that modification on the cell membrane cloaking on the nanoparticles is a promising strategy.

## 2 DIRECT MODIFICATION OF THE COATING CELL MEMBRANES

**FIGURE 1:**
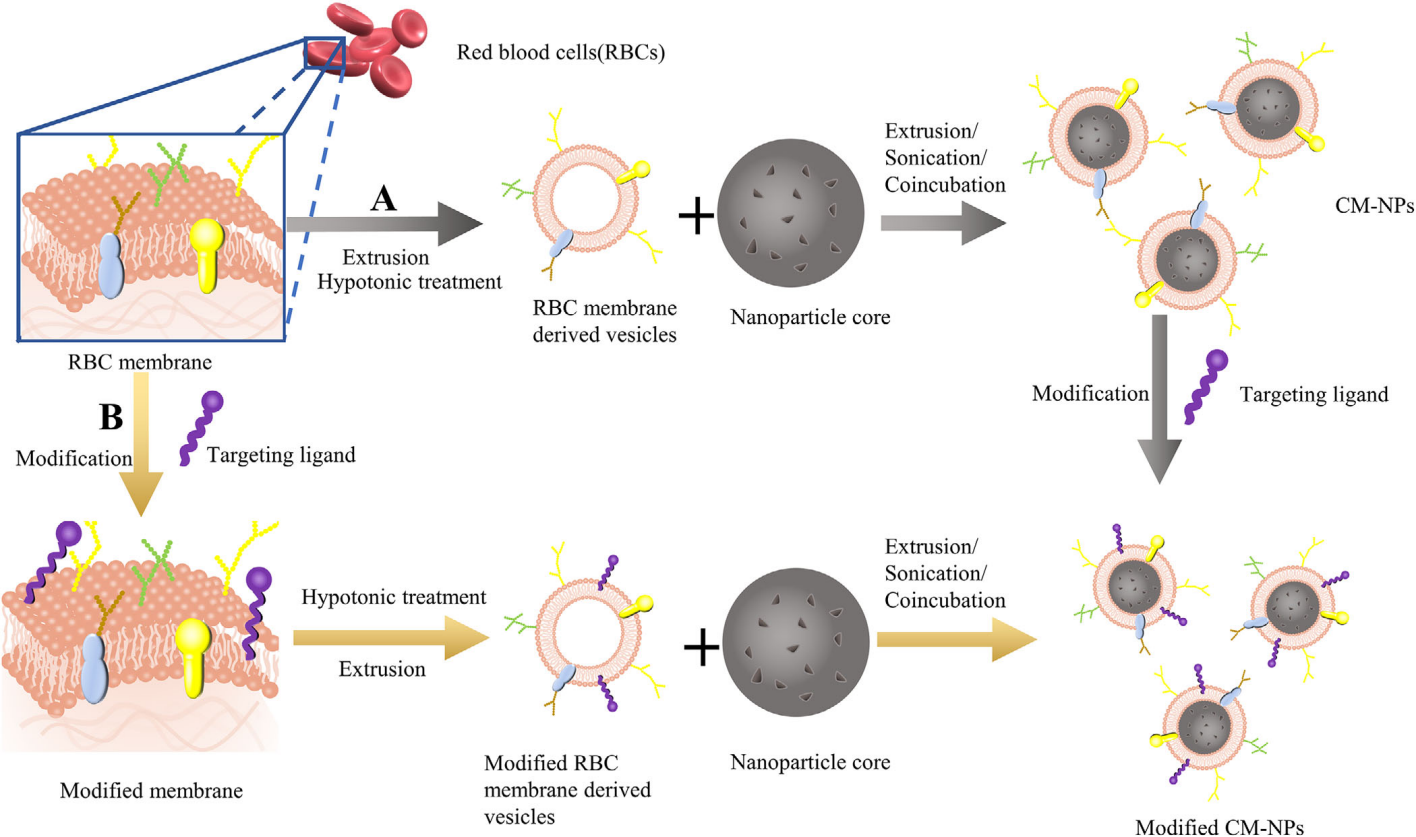
Preparation and modification of cytomembranes camouflaged nanoparticles. (A) The functional targeting ligands were directly modified on the membrane surface of CM‐NPs after the CM‐NPs had been successfully prepared. (b) The living cell membranes were modified before extraction

For the sake of realizing multifunctional cell membrane‐camouflaged nanoparticles, directly performing functional modification on the membrane surface of CM‐NPs seems to be a feasible and effective approach that a growing number of scholars pay attention to (Figure 1A). This is mainly achieved by integrating specific ligands into the surface of CM‐NPs to target the receptors overexpressed on the target cell surface or inserting the functional protein outside the lipid bilayers to penetrate the target sites and lessen the possibility of side effects.^30^ At present, there are three kinds of engineering strategies: noncovalent modification, covalent modification, and enzyme‐involved modification. Herein, we will discuss the preparation process and the experimental effect of these different strategies.

### 2.1 Noncovalent modification for cytomembranes camouflaged nanoparticles

**FIGURE 2:**
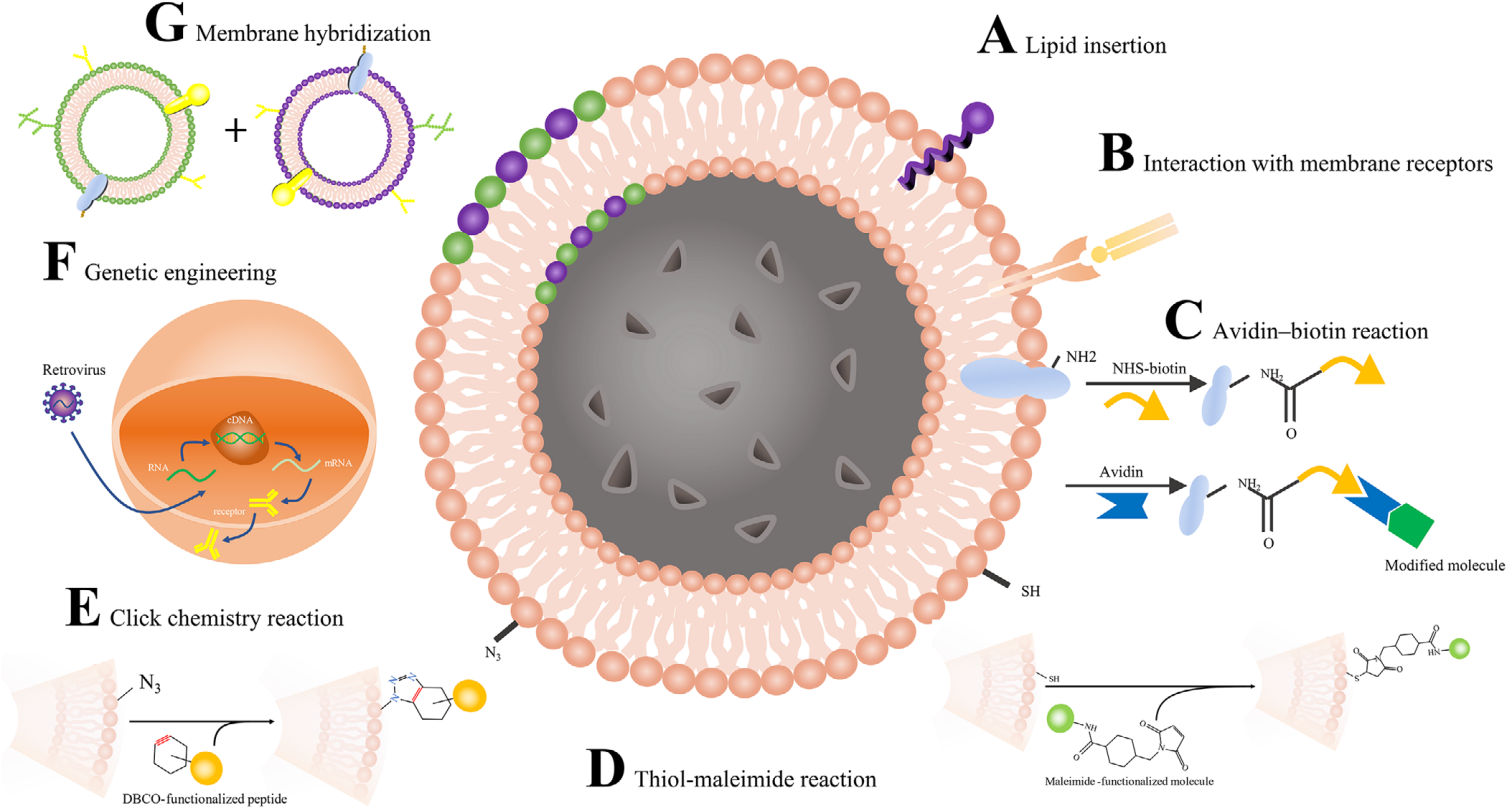
Strategies for CM‐NP modification. (A) Functional lipids can be spontaneously integrated into the phospholipid bilayers by hydrophobic interactions. (B) Several molecules can interact with membrane proteins, such as antigens and certain peptides bind to the receptors on the cytomembranes relying on the ionic bond and hydrophobic interactions. (C) In the avidin‐biotin reaction, biotins firstly anchor to the amino groups on cell membranes to construct the biotinylated CM‐NP. Then, the biotinylated groups conjugate with avidin/streptavidin anchored to the therapeutic molecules. (D) The therapeutic molecules conjugated to maleimide groups can link to the membranes via thiol groups. (E) N3 decorated on the membrane conjugates to the DBCO compound linked with therapeutic molecules by the copper‐free click chemical reaction. (F) Engineered cells express the desired products on the surface by transcription and translation of the gene. (G) Two different kinds of cytomembranes can be fused and the cytomembrane of hybrid cells can co‐expressed functional proteins derived from different cells

All strategies have their own inherent merits and demerits. Noncovalent modification is relatively more moderate and harmless to retain the activity of protein on the cell membrane surface.^31^ Lipid insertion is a simple but stable modification approach in common use, by which functional moieties linked with lipids can be spontaneously integrated into the phospholipid bilayers by hydrophobic interactions (Figure 2A).^32^ The functional molecules can acquire a higher binding force if they link with multiple hydrophobic interactions. On the other hand, the lipids inserted into the outer leaflets of membranes show a good stability and firm molecule attachment on cells.^31^ The cancer cell membrane‐coated nanoparticles with PEGylated phospholipid (DSPE‐PEG) inserted into the lipid bilayers constructed by Tian et al have indicated good properties of the structure.^33^ Before the process of lipid insertion, certain molecules should be activated to conjugate with the lipid portion. To acquire the red blood cell (RBC) membrane‐coated nanoparticles with hyaluronic acid (HA) integrated on the surface, Liu et al applied N‐hydroxysuccinimide (NHS) to modify HA to obtain the activated HA.^34^ It is best to avoid the existence of serum in the process of incubating the RBC membrane‐coated nanoparticles with modifying molecules, in case of those free proteins in serum can competitively bind to the lipid portion of modifying molecules, which restrain the efficiency of the molecules inserting into the cell membrane.^31^

In addition to lipid insertion, binding to the proteins on the surface of CM‐NPs is another noncovalent modification strategy (Figure 2B). For example, antigens and certain peptides can bind to the certain domains of membrane proteins relying on the ionic bond and hydrophobic interactions with the properties of high affinity and reversibility.^35^ However, the conjugation is generally random. The changes of functional domains may cause the proteins conjugated on the membranes to lose function. To avoid entirely membrane invalidation, fusing functionalized and innate membranes is an effective strategy.^36^

### 2.2 Covalent modification for cytomembranes camouflaged nanoparticles

Chemical approaches modify the cytomembranes surface of CM‐NP mostly via covalent bonds. Compared with hydrophobic interactions of noncovalent strategy, the covalent bonds offer more stable anchorage.^37^ It was found that the outer leaflets of cytomembranes exist mass activated primary amine groups which can react with activated carboxylic acid groups of therapeutic molecules to form amide bonds.^38^ In this chemical reaction, the carboxylic acid groups are supposed to convert into acyl chlorides, which are unstable intermediates tending to hydrolysis. The hydrolysis of acyl chlorides affects the productiveness. To solve this problem, NHS was introduced to modify molecules, increase stabilization of reaction by inverting carboxylic acid groups into relatively stable groups.^39^

Biotin‐avidin binding is another widely‐used approach to modify cytomembranes (Figure 2C). In this approach, Chai et al firstly anchored biotin to the cell membranes to construct the biotinylated CM‐NP. Then, the biotinylated groups conjugated with streptavidin anchored to the modifying molecules.^40^ However, due to the immunogenicity of avidin/streptavidin, biotin‐avidin binding can activate immune clearance, and it is not an appropriate approach to be applied to clinical therapy.^41^

Thiol–maleimide reaction is recently proposed. Researchers utilize the NHS‐PEG2‐maleimide, an impermeable linker to modify the cytomembranes of CM‐ NP (Figure 2D).^42^ The synthetic maleimide groups conjugate to the therapeutic molecules via thiol groups of molecules. This approach is suitable for modifying immunocyte membranes because the membranes of B cells and T cells possess massive thiol groups prone to undergo thiol–maleimide reaction.^43^

Zhang et al utilize click chemical reaction to construct therapeutic molecules‐modified antigen‐presenting cells (APCs) (Figure 2E).^44^ The dibenzocyclooctyne (DBCO) was anchored to the therapeutic molecules, T‐cell stimulatory signals co‐stimulatory ligand anti‐CD28 (αCD28), and peptide (SIINFEKL)‐loaded major histocompatibility complex class‐I (pMHC‐I). The successful anchor can be detected by the increased weight of modified molecules. They utilized intrinsic biosynthesis to decorate the leukocyte membrane with azide (N3). N3 conjugates to the DBCO compound by the copper‐free click chemical reaction.

However, since the covalent modification always lack specificity, some activated groups may react with the proteins expressed on the cytomembranes of CM‐NP, consequently leading to the inactivation of membrane proteins and impairing its original function.^31^

### 2.3 Enzyme‐involved modification for cytomembranes camouflaged nanoparticles

Since the insufficient steadiness of hydrophobic interactions and the possibility of impairing the intrinsic cytomembranes function in the covalent modification approaches, a more efficient and reliable strategy is anticipated. The enzyme‐involved modification offers a possible method with high selectivity, which introduces therapeutic molecules onto cytomembranes by an enzymatic reaction. Strictly speaking, enzyme‐involved reaction is also a kind of covalent modification. Sackstein et al introduced therapeutic molecules to the surface proteins CD44 of human mesenchymal stem cell by glycosyltransferase, enhancing the ability to target P/E selectins on target cells.^45^ Biotin ligase has been employed to catalyze the reaction of lysine residues on the cytomembranes and ketobintin.^46^ Sortase has also been utilized to introduce oligoglycine nucleophiles on the membrane proteins.^47^

Although the enzyme‐involved approaches obtain encouraging progress on the cytomembrane modification, it is difficult to utilize it to construct membrane‐engineered CM‐NPs. Each enzyme‐involved reaction demands the unique enzyme, which is hard to be separated and purified from living cells. Besides, it is tough to fully control the biochemistry reaction rate, which can be affected by multiple parameters, such as membrane proteins and reaction temperature.

## 3 ENGINEERING CELL MEMBRANES VIA CELL MODIFICATION

Lately, an increasing number of scholars have a disposition to engineer cell membranes at the cellular level (Figure 1B) rather than directly utilizing molecules to transform the phospholipid groups or proteins on the membranes of assembled CM‐NPs. There are several benefits in decorating the living cell membranes before extracting cytomembranes, compared to direct modification of CM‐NPs. Firstly, the separation of CM‐NPs with free molecules is a tough process consuming a lot of time and can ruin the functional membrane cloaks of CM‐NP. By comparison, segregating modified living cells and free uncombined molecules is uncomplicated. In case of retaining the functional membrane structure and saving time, modification on the living cell platform seems to be a more appropriate strategy. Secondly, the interaction of anchored molecules can enhance the formation of right‐side‐out orientation on the cytomembranes of CM‐NPs, which is vital to achieve multifunctional compounds. Thirdly, a portion of synthetic CM‐NPs demonstrate the inside‐out orientation of the coating membranes.^48^ This wrong construction can result in opposite orientation of functional domains, which may become dysfunctional. Modifying the membranes of living cells guarantees the correct orientation of the anchoring process.^37^

### 3.1 Genetic engineering techniques to modify cell surfaces

**FIGURE 3:**
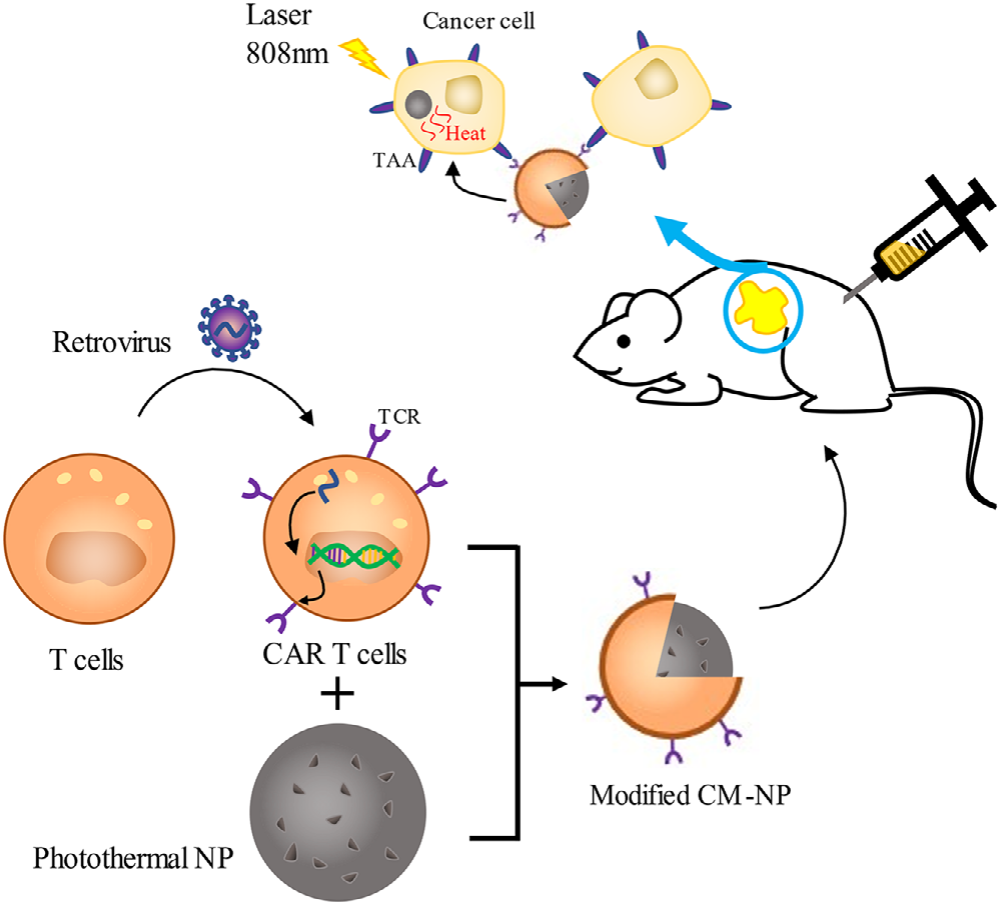
The tumor‐associated gene was integrated into the genome via transduction of retrovirus, and expressed CAR on membrane. The CAR‐T CM‐NPs targeted the TAAs expressed on cancer cells and released photothermal nanoparticles to eliminate tumor cells. Recreated from Ma et al.^50^

Genetic manipulation seems to be a feasible strategy of modification for CM‐NPs (Figure 2F). Engineered cells express the desired products on the surface by transcription and translation of the genes. T cells can be modified to target tumor‐associated antigens by introduction of the gene encoding artificial T cell receptors called chimeric antigen receptors (CAR). After transduction by retrovirus, CAR‐T cells expressing antigen receptors can provide bioengineered membranes to camouflage the inner cores.^49^, ^50^ These novel nanomaterials intend to combine the targeting capability of CAR‐T cells with the advantages of nanoscale cores. In vitro and in vivo experiments, novel CAR‐T cell membrane‐coated photothermal nanoparticles displayed the enhanced tumor targeting ability, minimal systematic toxicity, and excellent photothermal effect (Figure 3).^50^ It indicates that the CAR‐T cell membrane‐coated nanoparticle is a promising tumor therapy in the future. Bose et al constructed human adipose‐derived stem cells overexpressing C‐X‐C chemokine receptor type 4 (CXCR‐4) on the outer leaflet of cytomembranes as the derived cells of synthetic CM‐NPs.^51^

However, the procedure of genetic manipulation is cumbersome and tough to ensure stable expression of the target gene. So the application of genetic engineering is not much at present.

### 3.2 Production of novel bio‐coatings via membranes fusion

Besides modification on the membrane surface, a promising technique fusing two different kinds of cytomembranes has been developed (Figure 2G), which is supposed to mix the merits of different source cell membranes and refrain from its innate limitation of single membrane.^30^ The functional proteins are co‐expressed on hybrid cytomembranes. Erythrocytes possess the property of long circulation life because their membranes express the “self‐markers,” which contributes to immune escape, but they lack the targeting ability.^52^ Platelets possess the quality of targeting impaired blood vessels and accelerating adhesion, while their circulation time is relatively short.^53^ To solve this problem, RBC‐platelet hybrid membrane‐camouflaged nanoparticles were fabricated.^54^ With the fluorescent label, the erythrocytes and platelets membrane proteins were retained on the dual membranes at the ratio identical to the input, which indicates that membranes of two different sources fused successfully, and their inherent membrane proteins were retained respectively. It was demonstrated that the hybrid [RBC‐P]NPs possess good biocompatibility and multifunction with long circulation life.

**FIGURE 4:**
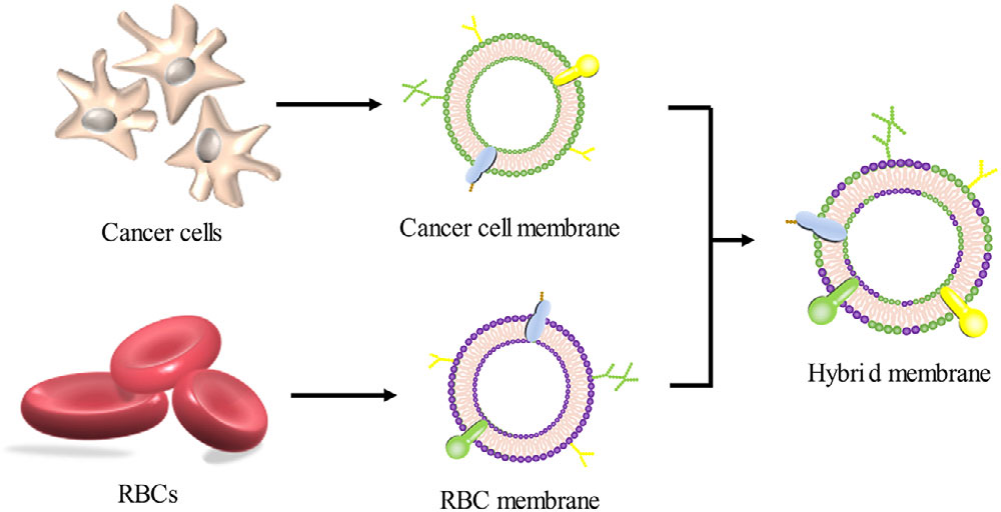
Membrane materials were respectively extracted from cancer cells and RBCs and then fused together. The hybrid membranes retained the membrane components derived from two different cells and played the inherent functions of two different kinds of cells

Cancer stem cells (CSC) obtain the specific cancer cell adhesion ability due to the homotypic targeting ligands expressed on their membranes.^55^ Platelet membrane surface possesses the “don't eat me” signals such as CD47, and, consequently, they can escape the immune attack.^56^ To design a tumor target compound with long circulation time, Bu et al fabricated a kind of novel CM‐NP by coating hybrid CSC‐P dual membranes onto iron oxide magnetic nanoparticles (MN).^57^ Confocal fluorescence microscopy images and western blot analysis indicate the [CSC‐P]MNs had been constructed successfully, and their surface markers were inherited. In contrast to the CSC‐MNs and platelet MNs, the [CSC‐P]MNs not only showed a milder immune response and lower IgG and IgM levels in vivo but also resulted in higher tumor accumulation and slower tumor volume growth. Cancer cell membranes are fine coating materials to construct cancer‐targeting CM‐NPs due to the excellent ability of homotypic targeting, drug delivery, and tumor penetration.^1^ Recently, fusing cancer cell membranes with erythrocyte membranes to construct the novel hybrid membranes is another strategy of enhancing circulation life (Figure 4).^58^, ^59^ The hybrid membranes retained the membrane components derived from different cells. Interestingly, raising the percentage of erythrocyte components increased the circulation time in vivo by refraining from cellular uptake of macrophages while raising the portion of cancer cell membranes components promoted the drug accumulation in the tumor sites. It was confirmed that the optimal protein weight ratio of erythrocyte and cancer cell membranes was 1:1 to balance the properties of homotypic targeting and immune escapability.^58^ Membrane fusion can not only target cancer cells but also enhance immunotherapy. Han et al successfully constructed the CM‐NP coated by hybrid Erythrocyte‐cancer cell membranes which elicited antigen responses, restricted tumor growth, and inhibited tumor recurrence and metastasis effectively.^60^

The fused membranes combine the merits of different source cells and effectively retain respective inherent membrane protein activities with this mild and biological approach. Taken together, this novel hybrid dual membrane‐camouflaged nanoplatform has a bright prospect for delivering drugs and multi‐functionalizing membranes.

## 4 FUNCTIONALIZED CELL MEMBRANE‐CAMOUFLAGED NANOPARTICLES FOR APPLICATIONS IN NANOMEDICINE

The cell membrane‐coated nanoplatform has been widely applied in the fields of drug delivery, imaging, detoxification, detection, and photoactivatable therapy due to the innate biological properties and excellent biocompatibility.^12^ Notably, the core particles play the important roles in the function of CM‐NPs and can be divided into two types: organic nanoparticles and inorganic nanoparticles. The choice of core particles is closely related to the purpose. Different nanoparticles have different properties.^61^, ^62^ Modification on cell membranes has been proved to be a great method for a large range of applications. Herein, we will discuss the applications of functionalized cell membrane‐camouflaged nanoparticles (Table 1).

**TABLE 1:** Application of functionalized cell membrane camouflaged nanoparticles

Application	Modified molecule	Membrane source	Core	Modification object	Modification strategies	Outcomes
Chemotherapy	rHuPH20	Erythrocyte	PLGA	Cell	Thiol‐maleimide reaction	Enhanced internalization by PC3 cells.^37^
	TRAIL	Platelet	Gelatin	CM‐NP	Lipid insertion	Anchored to the death receptors on the membranes of cancer cells, inducing cellular apoptosis.^67^
	RGD	Erythrocyte	Docetaxel	Cell	Avidin–biotin reaction	Enhanced targeting ability.^99^
	anti‐EGFR‐iRGD	Erythrocyte	PLGA	Cell	Thiol‐maleimide reaction	Enhanced the ability of specific tumor penetration.^69^
	^D^CDX	Erythrocyte	PEG	CM‐NP	Avidin–biotin reaction	Effectively penetrated across the blood‐brain barrier and showed excellent brain tumor‐targeting ability.^40^
	RGD	Erythrocyte	Fe_3_O_4_	CM‐NP	Thiol‐maleimide reaction	Boosted tumor uptake and minimized side effects of other organs.^101^
	anti‐EGFR‐iRGD	Erythrocyte	PLGA	CM‐NP	Lipid insertion	Facilitated the colorectal cancer targeting and antitumor ability.^68^
	DSPE‐PEG	Cancer cell	PLGA	CM‐NP	Lipid insertion	Overcame hypoxia‐induced chemoresistance of MCF‐7 cells.^33^
Photothermal therapy	Anti‐EpCam antibody	Erythrocyte	Au	Cell	Avidin–biotin reaction	Realized the selective targeting.^79^
	GPC3 recepter	T cell	silica	Cell	Genetic engineering	Combined excellent tumor targeting ability with photothermal effect.^50^
	NA	CSC‐ platelet	Fe_3_O_4_	Cell	Membrane hybridization	Possessed homotypic targeting ability with escaping from immune elimination.^57^
	NA	Erythrocyte‐cancer cell	Melanin	Cell	Membrane hybridization	Potentiated homogeneous targeting abilities with long circulation life, boosting pyrogen accumulation.^58^
	N_3_	T cell	PLGA	Cell	Click chemistry reaction	Significantly increase the photothermal therapeutic effect.^102^
	RGD	Bacteria	FeO	Cell	Genetic engineering	Improved the target‐binding specificity toward integrin positive cells.^103^
Photodynamic therapy	FA/TPP	Erythrocyte	UCNP	CM‐NP	Lipid insertion	Enhanced stealth ability and production of ^1^O2.^76^
Chemo/photothermal therapy	RGD	Platelet	Melanin/doxorubicin	Cell	Interaction with membrane proteins	Targeted the αvβ3 integrin and induced apoptosis.^36^
	HA	Erythrocyte	Prussian blue	CM‐NP	Lipid insertion	Differentially targeted the CD44 on the membranes of breast cancer cells.^34^
	NA	Erythrocyte‐cancer cell	CuS	Cell	Membrane hybridization	Potentiated homogeneous targeting abilities with long circulation life.^59^
Immunotherapy	Mannose	Erythrocyte	PLGA	Cell	Lipid insertion	Enhanced IFN‐γ secretion and CD8+T cell response.^89^
	pMHC‐I/αCD28	Antigen‐presenting cell	MNC	Cell	Click chemistry reaction	Stimulated and expanded CTL effectively.^44^
	Surface‐layer protein	Cancer cell	HPAD	CM‐NP	self‐assembly	Protected the antigen on the membrane and enhancing T‐cell proliferation.^87^
	Mal	Bacteria	CpG/PC7A	CM‐NP	Reaction with primary amines	Enhanced antigen released from cancer cells uptake.^39^
	Mannose	Cancer cell	PLGA	CM‐NP	Lipid insertion	Showed increased DC uptake and high‐performance stimulation of DC maturation.^104^
	NA	Erythrocyte‐cancer cell	PLGA	Cell	Membrane hybridization	Elicited antigen responses and inhibited the tumor growth in vivo.^60^
	NA	Bacteria‐cancer cell	HPDA	Cell	Membrane hybridization	Enhanced the antitumor efficacy toward melanoma.^105^
	SIRPα variants	macrophage	MNC	Cell	Genetic engineering	Blocked the CD47‐SIRPα pathway and triggered immune responses.^106^
Imaging	FA	Erythrocyte	UCNP	Cell	Lipid insertion	Enhanced tumor imaging in vivo.^96^
	DiR	Erythrocyte	PCL	Cell	Lipid insertion	Enhanced visualization of fluorescence probe in vivo.^97^
	FA/AS1411	Erythrocyte	PLGA	Cell	Lipid insertion	Enhanced targeting ability.^107^
Others	CXCR4‐receptor	hASC	PLGA	Cell	Genetic engineering	Targeted injured tissues and potentiated the nanoparticle penetration across endothelial cells to cure severe hindlimb ischemia.^51^
	rt‐PA	Platelet	PLGA	CM‐NP	Thiol‐maleimide reaction	Enhanced thrombolytic therapy with a low bleeding risk.^98^
	DBCO‐Ab	Leukocyte	Fe_3_O_4_	CM‐NP	Click chemistry reaction	Showed high‐performance recognition and enrichment of circulating tumor cells.^108^
	T807/TPP	Erythrocyte	HSA	CM‐NP	Lipid insertion	Promoted sustained drug release in the brain.^109^

Abbreviations: αCD28, anti CD28; CDX, peptide FKESWREARGTRIERG; CSC, cancer stem cell; CXCR4, C‐X‐C chemokine receptor type 4; DBCO, dibenzocyclooctyne; DiR, cyanine dye 1,1‐dioctadecyl‐3,3,3,3‐tetramethylindotricarbocy‐anine iodide; EpCam, epithelial cell adhesion molecule; FA, folic acid; HA, hyaluronic acid; hASC, human adipose‐derived stem cell; HPAD, polymer DOX/polytheyleneimine‐modified (2‐hydroxypropyl)‐γ‐cyclodextrin; HPDA, hollow polydopamine; HSA, human serum albumin; Mal, maleimide group; MNC, magnetic nanocluster; PCL, poly(caprolactone)‐ester endcap polymer; PLGA, poly (lactic‐co‐glycolic acid); pMHC‐I, peptide‐loaded major histocompatibility complex class‐I; RGD, peptide Arg‐Gly‐Asp; rHuPH20, human recombinant hyaluronidase, PH20; rt‐PA, recombinant tissue plasminogen activator; TPP, triphenylphosphonium; TRAIL, tumor necrosis factor‐related apoptosis‐inducing ligand; UCNP, upconversion nanoparticle.

### 4.1 Chemotherapy

Chemotherapy is one of the most widely applied traditional cancer treatments in clinical application. Traditional chemotherapy inhibits tumor proliferation by cellular toxicity. Due to the deficiency of bioavailability and systemic toxicity of the classic strategy, the novel carriage formulations have been desired to improve the drug efficiency and reduce side effects.^63^ Poly (lactic‐co‐glycolic acid) combines the high drug loading capacity with biocompatibility, so it is a suitable choice to use it as the carriers of chemotherapeutic drugs.^54^ The membrane‐coated, chemotherapeutic drug‐loaded nanoparticles have been the prospective therapeutic compounds, and the manipulable membranes can obtain additional function by artificial modification.

HA is an essential component to compose the extracellular matrix.^64^ It has been observed that the expression of HA is increased around most tumor tissues, which inhibits the chemotherapeutic drug penetration and uptake.^65^ Zhou et al conjugated rHuPH20, a recombinant human hyaluronidase, on the membranes of RBCM‐NPs.^37^ The PH20‐RBCM‐NPs displayed more apparent binding and internalization by PC3 cells under fluorescence imaging compared with RBCM‐NPs. Besides, the circulation life of PH20‐RBCM‐NPs was extended. One possible reason was that the PH20 conjugation decreased the formation of RBCM‐NPs with an inside‐out orientation during extraction as the conjugation prevented the therapeutic cores from being coating by the outer leaflet of erythrocyte membranes.

Red blood cell and platelet membranes are most widely used cladding materials of therapeutic nanoparticles for extensive purposes as the structures without the nucleuses and organelles are convenient to acquire and decrease the interference of the intracellular matrix.^66^ For instance, Chai et al successfully fabricated the brain‐targeting ^D^CDX‐RBCNPs/doxorubicin (DOXS) using modified erythrocyte membranes.^40^ They displayed a great property to cross the blood‐brain barrier as the modifying ligand ^D^CDX possesses an efficient ability to bind to the nicotinic acetylcholine receptors on the membranes of brain endothelial cells. Hu et al fabricated platelet‐mimicking nanoparticles loaded with DOX.^67^ They decorated the platelet membranes with TRAIL, a ligand targeting the death receptors on the surface of cancer cells. It was indicated that the novel nanovesicles had a great affinity to targeted cells and enhanced cellular apoptosis in lung tumor tissues.

There is a novel strategy that modified the erythrocyte membranes with anti‐EGFR‐iRGD proteins, the bispecific recombinant proteins which combined the EGFR antibodies and iRGD peptides.^68^, ^69^ The iRGD peptides can bind to the αvβ3/αvβ5 integrin receptors overexpressed on the surface of cancer cells and enhance the vascular permeability.^70^ EGFR antibodies target multiple tumor tissues, especially in colorectal cancer.^71^ It was indicated that the modification of these bispecific recombinant ligands brought RBC‐NPs with bispecific targeting abilities and facilitated the tumor accumulation and cellular toxicity of chemotherapeutic drugs.

### 4.2 Phototherapy

Phototherapy is a noninvasive and practical therapeutic strategy for cancer patients. It is induced by laser irradiation, including PDT and PTT.^72^, ^73^ Owing to the magnetic and photothermal performance, metals are the preferred material for core nanoparticles.^74^ Singlet oxygen (^1^O_2_) is the active therapeutic molecule in PDT, which causes mitochondria damage to destroy tumor tissues and vasculature. The ^1^O_2_ is converted from ground‐state molecular oxygen (^3^O_2_) by light‐triggered photosensitizer (PS).^75^ Ding et al manipulated the RBCM‐camouflaged PS‐loaded biomimetic PDT agents with FA/TPP dual‐targeting molecules.^76^ Based on the RBCM‐NPs with natural oxygen‐carrying capacity, excellent biocompatibility, and stealth ability to escape from the reticuloendothelial system, the modified FA/TPP targeted tumor tissues and facilitated dye‐labeled NPs internalization. Besides, the 30‐day mortality and tumor volume were lower than undecorated groups.

PTT has been promising and rapidly improving cancer treatment with low systemic side effects.^77^ It utilizes the heat ablation effect induced by light‐absorbing agents under near‐infrared (NIR) irradiation to diminish the tumor tissues.^78^ Engineered RBC membrane‐coated gold nanocages were fabricated.^79^ Its membranes were decorated with anti‐EpCam antibodies, the antibodies targeting the epithelial cell adhesion molecules which are overexpressed on the membranes of several cancer cells.^80^ The results displayed that the anti‐EpCam antibodies‐modified group induced more photothermal cores uptake by 4T1 cancer cells and more dead cancer cells observed under the photothermal cytotoxicity images. Besides, Jiang et al fabricated the fused erythrocyte‐cancer cell membrane coated melanin‐loaded nanoparticles. The study showed that the temperature of tumors was elevated under NIR, and tumor weight was decreased, and it is best under the protein weight ratio of 1:1. It is indicated that the novel nanoparticles possessed the great ability of homologous targeting and long circulation time.^58^

A novel cancer treatment that utilizes membrane‐coated nanotechnology to co‐deliver light‐absorbing agents and chemotherapy drugs has drawn much attention.^81^, ^82^ The platelet membranes were coated onto the mesoporous silica nanoparticles (MSNs) loaded with DOX, which was designed as drug delivery vehicles for chemo/PTT. To enhance the ability to target cancer cells, Jing et al modified RGD peptides on the membranes.^36^ The platelet membrane‐coated MSNs produces a multipronged effect under the NIR laser, including reduction of the cancer cell viability and inhibition of tumor metastasis. The modified RGD peptides could effectively target the αvβ3 integrins overexpressed on the cancer cells to facilitate drug infiltration and tumor ablation under NIR.

### 4.3 Immunotherapy

**FIGURE 5:**
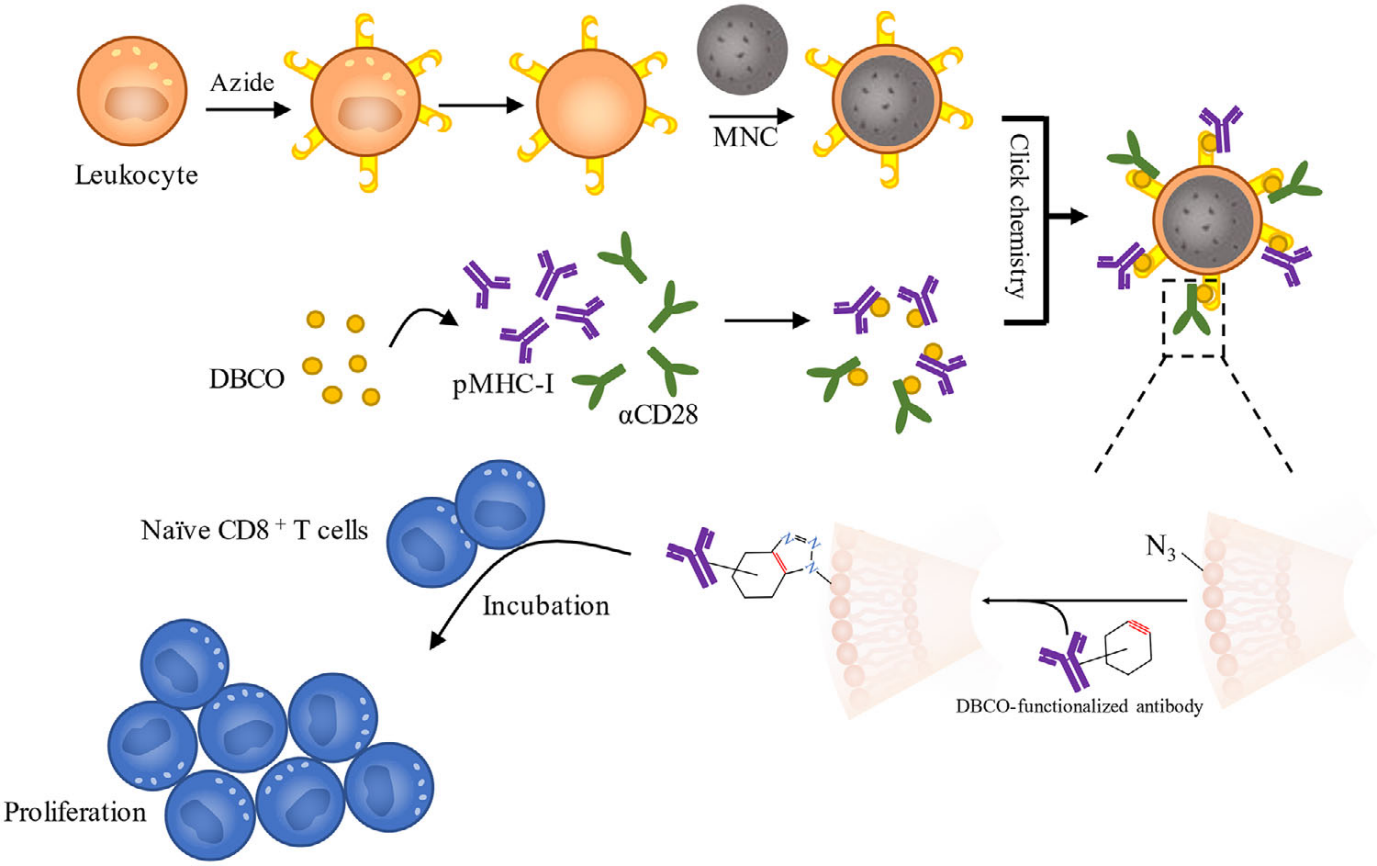
The leukocyte membrane was pre‐engineered with azide (N3) via intrinsic biosynthesis and metabolic incorporation of phospholipids. Dibenzocyclooctyne (DBCO)‐modified T‐cell stimuli (such as anti‐CD28 and pMHC‐I) could be decorated through copper‐free click chemistry. The biomimetic aAPCs efficiently expanded and stimulated naïve CD8+ T cells ex vivo. Recreated from Zhang et al.^44^

Cancer immunotherapy utilizes inherent immune response to eliminate cancer cells and restrain metastasis.^83^, ^84^ The modification of cell membrane‐coated nanoparticles has been applied to provide a controllable, targetable, and efficient immunotherapy approach with low systemic side effects.^85^, ^86^ Wu et al manufactured S protein‐modified cancer cell membrane‐camouflaged nanoparticles loaded with DOX.^87^ The S proteins extracted from *Lactobacillus helveticus* are inherent adjuvants and protect the antigens expressed on the cancer cell membranes, potentiate cytokine secretion, and induce an enhanced antitumor immunity. The membranes of certain bacteria express pathogen‐associated molecular patterns so that they possess the properties of capturing the neoantigens and facilitating dendritic cell (DC) maturation.^88^ It was indicated that the modified maleimide groups on the surface of bacteria membrane‐coated nanoparticles enhanced tumor‐specific antigen uptake by DC, which boosted DC maturation and antigen presentation.^39^ In another study, mannose was decorated on the surface of erythrocyte membrane‐coated polymeric nanoparticles to construct nanovaccine for antitumor immunity induction.^89^ It was indicated that the decorated mannose effectively induced the vaccine delivery to the lymph nodes and enhanced the antigen uptake to boost DC maturation.^89^, ^90^ Besides, there is a modification strategy that directly modify antigens on the artificial APCs (aAPCs).^44^ Zhang et al fabricated biomimetic magnetosomes coated with azide‐engineered leukocyte membranes, and the co‐stimulatory ligand anti‐CD28 and pMHC‐I were decorated on the membrane surface through copper‐free click chemistry (Figure 5). The artificial anti‐CD28 and pMHC‐I displayed great properties to activate naïve CD8^+^ T cells ex vivo. After transfusion to the tumor‐bearing mouse, the reinfused cytotoxic T lymphocytes eliminated cancer cells effectively. Recently, Cheng et al fabricated an artificial nanovaccine, denoted “mini DC,” which is the IL‐2‐loaded PLAG nanoparticle coated by membranes of cancer antigen‐primed DC.^91^ The functional membrane proteins (such as MHC‐Ⅱ and CD28) expressed on the mature DC surface could stimulate cancer specific immune responses and caused the efficient inhibition of ovarian tumor growth and metastasis.

Taken together, these studies indicated there is great potential in surface modification of membrane‐coated nanoparticles for antigen delivery in cancer immunotherapy.

### 4.4 In‐vivo imaging

**FIGURE 6:**
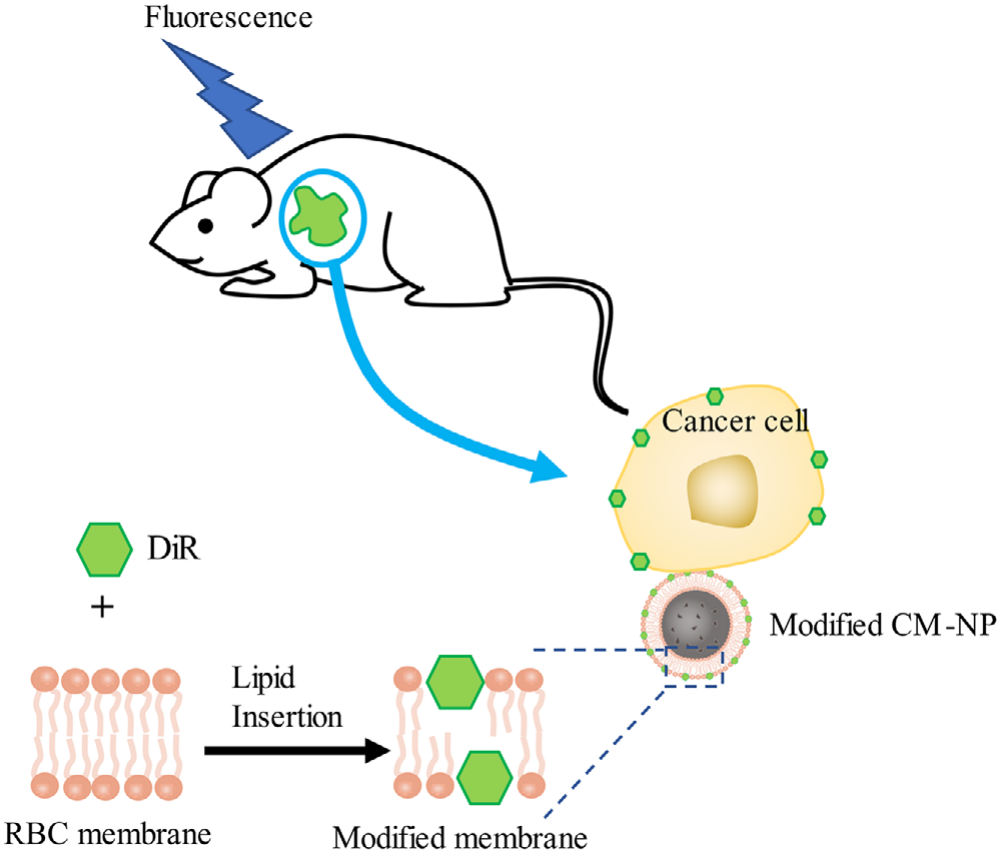
Cyanine dye DiR was inserted into bilayer lipid membrane by noncovalent interactions, and modified CM‐NPs were absorbed by tumor cells. Under near‐infrared laser, DiR incorporated into CM‐NPs showed increased fluorescence. Owing to the accumulation of DiR in cancer cells, in vivo imaging and biodistribution of tumors could be realized. Recreated from Su et al.^97^

In addition, exploiting live cell membranes as the cloaks to be coated onto contrast agent nanoparticles has been employed in biomedical imaging of tumors with multiple advantages, such as long circulation time, less systemic side effect, and great tumor accumulation.^92^, ^93^, ^94^, ^95^ In recent years, surface modification has been applied in fluorescence imaging. Folic acid (FA), the selective tumor marker ligand anchoring to its receptors overexpressed on the multiple cancer cells, was decorated on the membranes of erythrocyte membrane‐coated fluorescence imaging nanoparticles.^96^ The FA‐functionalized nanoparticles showed a great property of high imaging agent content in the tumor sites without systemic poisonousness. Except for modification with tumor‐targeting ligands, another strategy that NIR dye is inserted into the membrane shells has also been applied.^97^ It was indicated that the NIR dye anchored on the CM‐NPs via noncovalent interaction elongated 12.3‐folder circulation life than that of the free dye and facilitated the intracellular uptake. Thanks to the accumulation of NIR dye in cancer cells, Li et al realized in vivo imaging and biodistribution of tumors (Figure 6).

The membrane modification of CM‐NPs has been exploited in not only anti‐tumor treatment but also other fields. For instance, Bose et al designed human adipose‐derived stem cell membrane‐coated nanoparticles modified by CXCR4‐receptor. The modified CM‐NPs could target injured tissues and potentiated the nanoparticle penetration across endothelial cells to cure severe hindlimb ischemia. Xu et al designed nanoplatelets modified by rt‐PA. The artificial nanoplatelets enhanced the thrombolytic therapy with a low bleeding risk.^51^, ^98^ Overall, modification on the membranes to functionalize CM‐NP is a promising strategy, which has been widely used.

## 5 CHALLENGES AND LIMITATIONS

Although membrane engineering technology of CM‐NP has displayed moderately satisfactory outcomes in various fields, this technology is in its infancy stage. Up to now, these nanoscale therapeutics are still in laboratory research phases. To make a transition from bench to clinical bed, several technical challenges need to be solved.

### 5.1 What are the difficulties

First, membrane modification technology is still immature. The complexity and low reproducibility of the preparation process restrict the scale of preparation.^21^

Second, although a variety of modification strategies have been developed, the reaction parameters in different experiments are various, such as the reaction temperature and substrate concentration. Necessary criteria are demanded. When it comes to membrane modification, we must realize that cytomembranes are a portion of living entities. In cell functionalization, the appropriate reaction conditions should be controlled, and the reagents that damage cell activities are forbidden to use. However, there is a lack of appropriate judgment basis to select these conditions to improve the modification efficiency.

Third, the method to determine whether membrane modification is successful is very limited, mainly through membrane potential.^99^ Particle size detection and morphology observation are hard to differentiate small modified molecules. Western blot analysis can merely distinguish the composition of the modified membrane and the original cell membrane. It is difficult to evaluate whether the cytomembrane activity is impaired after the modification. It is necessary to design a way to visualize the membrane modification process.

Last but not the least, the stability of the membrane in this engineering process is difficult to guarantee. It is evitable that the modification process interferes with the surface activity and blocks membrane proteins. In a long time of reaction and preservation process, it is difficult to keep CM‐NPs away from virus and pyrogen contamination, and the membrane proteins tend to be denatured by the potential immune response. Overall, there are still some defects in the safety and effectiveness of membrane modification technology. Exploring more efficient and standardized modification strategies is the key to realize large‐scale preparation.

### 5.2 What are the limitations

Due to restrictions from technology and methodology, no membrane engineering strategy is perfect. Each modification method has its inherent benefits and drawbacks. The noncovalent modification offers preferable protection of membrane protein activity, while the noncovalent interaction is not firm enough compared with covalent bonds.^100^ Covalent bonds anchor functional molecules on the membranes solidly, but it is easy to compromise the membrane protein profile and sacrifice the natural function of cytomembrane due to the conventional chemical reaction. Limited by the technology of production, separation, and purification, enzyme‐involved modification is difficult to be applied to the optimization of CM‐NP. Several studies have shown that the same modified molecules could be attached to the cytomembrane by different strategy (for instance, the successful binding of RGD to RBC membranes can be achieved by thiol‐maleimide or avidin–biotin reaction^99^, ^101^), but it is unknown which approach is better for retaining membrane protein activity and proper orientation with the firm linkage. Moreover, the effect of different types of nanoparticles on the process of membrane engineering cannot be ignored. Studies on verifying effects of different modification strategies are needed.

Moreover, there is still a lack of consensus on whether to modify on the platform of the living cell membranes or the constructed CM‐NPs. Cell‐based modification introduces only a small portion of total modified content into nanovesicles, which reduced the utilization efficiency of reagents and materials. By comparison, direct functionalization on CM‐NPs guarantees all modified molecules are situated at outer leaflets of vesicles.^15^ However, as previously described, modifying on the living cells has superiority in separation and formation of right conformation. It seems that each strategy has its own limitation.

## 6 CONCLUSIONS AND PROSPECTS

The cell membrane‐camouflaged nanoparticles have been feasible nanoplatforms with great biocompatibility. The membrane modification of CM‐NPs nowadays is one of the research focuses to overcome the inherent limitation of the natural membrane. Multiple studies have successfully modified the membranes of CM‐NPs to confer extra properties of nanoparticles in cancer treatment, such as chemotherapy, PTT, PDT, immunotherapy, and imaging. Cell membrane‐based cloaks wipe out the potential toxicity and immunogenicity of nanoparticles, and the modified molecules endow the CM‐NPs with more powerful capabilities, such as tumor targeting, cell internalization, and enhanced immune response.

At present, the preparation and modification of membrane‐coated nanoparticles are still limited to a small scale in the laboratory. For the sake of expanding the output and reducing the cost, we still need to optimize the processing procedure. In the future, we should work to reach a consensus on the parameters of modifying process and develop a modifying strategy that guarantees not only the efficiency of modification but also the activity of membrane protein.

In conclusion, the membrane modification technology of CM‐NPs is still in its infancy. There are still many problems to be overcome, but it cannot be ignored that the membrane modification has been confirmed to enhance the therapeutic and diagnostic effect of CM‐NPs. We have a strong sense to believe that the membrane modification of CM‐NPs provides a wide range of possibilities for the research and application of nanomedicine.

## CONFLICT OF INTEREST

The authors declare that they have no known competing financial interests or personal relationships that could have appeared to influence the work reported in this paper.

## AUTHOR CONTRIBUTIONS

Minghai Zhang, Shanshan Cheng, and Yu Wang participated in the conception and design. Minghai Zhang and Shanshan Cheng draft the manuscript. Yue Jin, Nan Zhang, and Yu Wang participated in the editing and revision of the manuscript. All authors have reviewed and approved the manuscript prior to submission.
